# Muscle UCP-3 mRNA levels are elevated in weight loss associated with gastrointestinal adenocarcinoma in humans

**DOI:** 10.1038/sj.bjc.6600074

**Published:** 2002-02-01

**Authors:** P Collins, C Bing, P McCulloch, G Williams

**Affiliations:** Gastroenterology Unit, University Hospital Aintree, Longmoor Lane, Liverpool L9 7AL, UK; Diabetes and Endocrinology Research Group, Department of Medicine, University of Liverpool, Liverpool L69 3GA, UK; Division of Surgery, University Hospital Aintree, Longmoor Lane, Liverpool L9 7AL, UK

**Keywords:** uncoupling proteins, cachexia, muscle

## Abstract

The mitochondrial uncoupling proteins-2 and -3 are putative mediators of thermogenesis and energy expenditure. We measured the mRNA levels of uncoupling proteins-2 and -3 in skeletal muscle from 12 gastrointestinal adenocarcinoma patients, of whom six had stable weight and six had lost 2–18 kg, and from six healthy controls undergoing elective surgery. Uncoupling proteins-3 mRNA levels were significantly higher in the muscle of the cancer patients with weight loss (2.2±0.47 arbitrary units) compared both with controls (0.39±0.20) and with cancer patients who had not lost weight (0.47±0.23; *P*<0.02). Uncoupling proteins-2 mRNA levels did not differ significantly between groups. Elevations in muscle uncoupling proteins-3 activity may enhance energy expenditure and this in turn could contribute to tissue catabolism.

*British Journal of Cancer* (2002) **86**, 372–375. DOI: 10.1038/sj/bjc/6600074 www.bjcancer.com

© 2002 The Cancer Research Campaign

## 

Some degree of weight loss occurs in over 80% of cancer patients, and is strongly associated with poor outcome and early death (Puccio and Nathanson, 1997; De Wyes *et al*, 1980). The aetiology of weight loss in malignancy is multifactorial and may include increases in circulating cytokines, reductions in food intake, and alterations in metabolism (Tisdale *et al*, 1996). Resting energy expenditure is increased in some animal and human cancers, and is thought to contribute to weight loss (Bosaeus *et al*, 2001). The sites and mechanisms of excessive heat production in human cancer cachexia are however unknown.

The uncoupling proteins (UCPs) are a family of mitochondrial membrane proteins. UCP-1 is restricted to brown adipose tissue (BAT) where it uncouples oxidative phosphorylation and generates heat instead of ATP (Klaus *et al*, 1991). Other UCPs include UCP-2 (found in many tissues) and UCP-3 (expressed in BAT and skeletal muscle) (Boss *et al*, 1997; Fleury *et al*, 1997). Both have uncoupling activity *in vitro* and may mediate the increased energy expenditure implicated in energy balance. Transgenic mice over-expressing UCP-3 in muscle are lean despite exhibiting hyperphagia; oxygen consumption in the transgenic animals was increased by 91% indicating an increase in metabolic rate (Clapham *et al*, 2000). The exact roles of the various UCP isoforms *in vivo* are still debated and are reviewed elsewhere (Ricquier and Bouillaud, 2000).

We recently found increased UCP-2 and -3 mRNA levels in the muscle of mice with severe cachexia due to the MAC-16 (Bing *et al*, 2000) This non-metastasizing adenocarcinoma is histologically similar to human gastrointestinal tumours and causes profound weight loss despite only modest reductions in food intake. Here, we aimed to determine whether UCP-2 and -3 expression was similarly up-regulated in human cancer.

## METHODS

### Patient characteristics

We studied 12 patients with histologically diagnosed adenocarcinoma of the GI tract undergoing elective laparotomy. Subjects were excluded from the study if there was evidence of intercurrent infection at the time of surgery, or mechanical obstruction of the gut. Six had weight loss of 2–18 kg over a period of 4–12 weeks prior to surgery, as documented in medical notes, while the remainder had stable weight documented on at least two occasions over a similar period. Weights were measured on a single set of scales in the surgical outpatient clinic. All patients were operated during a weekly list to minimize the differences in fasting times prior to sample collection. Controls were undergoing laparotomy for a benign disease (Table 1

**Table 1:**
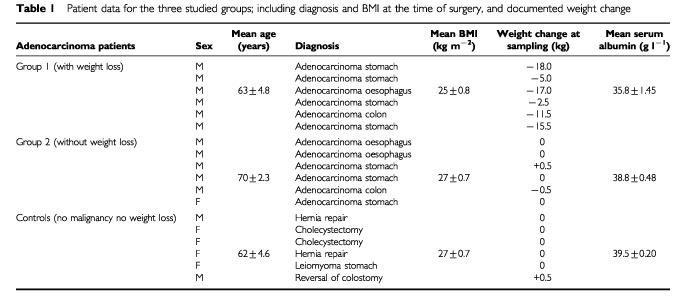
Patient data for the three studied groups; including diagnosis and BMI at the time of surgery, and documented weight change

) and had a documented stable weight for at least 8 weeks prior to surgery.

Fully informed and written consent was obtained in all cases and the study protocol was approved by the Sefton Ethics Committee.

### Tissue collection and processing

At laparotomy, samples of rectus abdominus muscle (approximately 10 mg) were collected and immediately placed in storage solution (RNAlater® Ambion Inc., Austin, TX, USA) to stabilize RNA pending transport to the laboratory and storage at −20°C. After collection, all samples were processed at the same time using a commercial spin column kit to isolate total mRNA (Neucleospin® Clontech Labs Inc., CA, USA). The amount and purity of isolated RNA was calculated using spectrometry at 260/280 nM.

### Measurement of UCP-2 and -3 mRNA

Semi-quantitative RT–PCR was performed to calculate amounts of UCP-2 and -3 mRNA, relative to the housekeeping gene, hypoxanthine phosphoribosyl transferase (HPRT). One ng of total RNA was added to 50 μl of PCR mixture containing UCP-2 or -3 specific primers (0.2 μM each of forward and reverse), reverse transcriptase, DNA polymerase, MgCl_2_ (1.5 mM) (OneStep® Abgene, Epsom, Surrey, UK). A further 1 ng sample was added to an identical PCR mixture containing HPRT-specific primers. Preliminary experiments identified RNA template concentrations, which yielded a product within a linear range for both HPRT and the UCPs, under the standard conditions defined below, and PCR products were sequenced to ensure specificity.

cDNA synthesis and PCR were completed in a single reaction: 30 min at 47°C for first-strand generation, inactivation for 2 min at 95°C, followed by 30 cycles (20 s at 94°C, 30 s at 56°C, 1 min at 72°C) and a final extension step at 72°C for 5 min. After completion, a 5 μl aliquot of the HPRT reaction was mixed with 10 μl of the UCP-2 or -3 reaction and the resulting mixture subjected to electrophoresis on a 1% agarose gel, stained with ethidium bromide. Bands were visualized and quantitated using a digital image analysis system (Kodak 1-D Eastman Kodak Co, MA, USA). Optical density was calculated for the two products and the results expressed as a ratio of UCP-2 product to HPRT product. A negative control (without reverse transcriptase) was run with each experiment to ensure the absence of DNA contamination.

## RESULTS

There were no significant differences in age between the three groups (Table 1). Most of the cancer group were male, while three of the control group were female. The origins of the adenocarcinomas were similarly distributed in the cancer patients with or without weight loss. In the cancer with documented weight loss group, this ranged from 2–18 kg. BMI and serum albumin were significantly lower in the cancer patients with weight loss than in both other groups.

Table 2

**Table 2:**
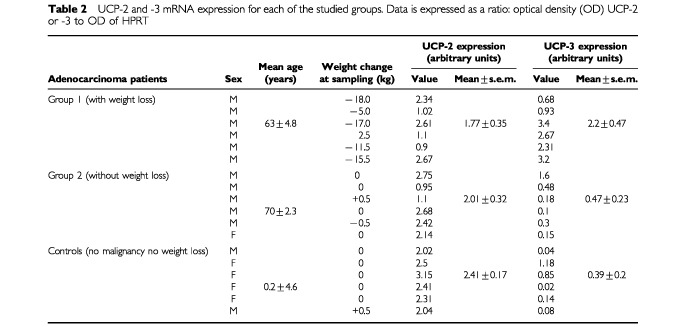
UCP-2 and -3 mRNA expression for each of the studied groups. Data is expressed as a ratio: optical density (OD) UCP-2 or -3 to OD of HPRT

shows the levels of UCP-2 and -3 mRNA, expressed as ratios to HPRT mRNA; these data are expressed graphically in Figure 1

**Figure 1:**
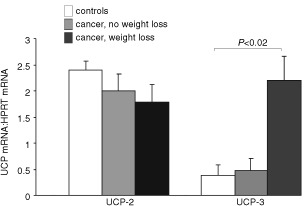
Mean UCP-2 and UCP-3 levels for the studied groups. Data is expressed as a ratio: optical density (OD) UCP-2 or -3 to OD of HPRT ±s.e.m.

. UCP-2 mRNA levels were similar among all three groups. Mean UCP-3 levels, however, were over five times higher in the cancer patients with weight loss group than in controls. In the cancer without weight loss group, mean UCP-3 levels were similar to controls.

## DISCUSSION

This is the first demonstration of up-regulation of UCPs in human cancer associated weight loss. We have shown that UCP-3 mRNA levels are increased in muscle only when weight loss is associated with cancer. UCP-2 mRNA levels in muscle seems unaffected by cancer either with or without weight loss. This finding correlates with that previously demonstrated in the MAC-16 model of cancer cachexia (although in this model UCP-2 was also increased in skeletal muscle compared with non tumour-bearing controls).

Our data suggests that UCP-3 in skeletal muscle might either contribute to weight loss, or be up-regulated as a consequence of metabolic changes brought about by tissue loss. It is well recognized that energy expenditure is increased in cancer often despite reduced energy intake, and that it seems directly related to body cell mass (Simons *et al*, 1999). That this increase in energy expenditure is directly related to the tumour effect is evidenced by the fact that removal of tumour ensures prompt restoration of normal metabolism (Luketich *et al*, 1990). UCP-3, when overexpressed in the skeletal muscle of transgenic mice results in a lean phenotype, with increased energy expenditure compared to controls (Clapham *et al*, 2000). Over expression of UCP-3 in the skeletal muscle of human cancer patients might contribute to increase energy expenditure and thus to weight loss.

Of course the fact that a protein associated with an increase in energy expenditure is over expressed in a situation where energy conservation would seem more appropriate, appears at first to be counterintuitive. However one of the putative roles of UCP-3 *in vivo* is the facilitation of lipid substrate utilization. Certainly lipid infusions result in increased skeletal muscle UCP-3 levels both *in vivo* and *in vitro* (Hwang and Lane, 1999; Samec *et al*, 1999). Lipid dysregulation occurs in cancer associated weight loss, with increases in lipolysis (caused by cytokines and tumour derived lipolytic factors) off-set by increases in lipid utilization so that plasma free fatty acid levels may remain normal or only slightly elevated (Younes *et al*, 1990). The role of UCP-3 may be to assist in the utilization of excess FFA as fuel in the muscle or to prevent possible harmful effects of the intermediates of FFA metabolism. Unfortunately in this study we were unable to obtain fasting FFA levels in enough of the subjects studied to enable a statistically meaningful correlation with UCP-3 levels. In our animal model, however UCP-3 mRNA levels correlated well with plasma FFA levels and there is no evidence to suggest that this would not be the case in humans.

If the up-regulation of UCP-3 in skeletal muscle in human cancers does result in increased energy expenditure and result in weight loss, this offers potential new targets for therapeutic intervention. Indeed, this observation may not be confined to weight loss associated with malignancy, but may also occur in other wasting conditions such as, HIV disease, rheumatoid and cardiac failure. Unfortunately due to the difficulty in obtaining laparotomy specimens from these groups we were unable to address this specific issue in the current study, but aim to investigate it at a future date. If mechanisms could be found to block the up-regulation of UCP-3 in malignancy and this resulted in a reduction in metabolic rate, weight stabilization may occur. Cachexia is one of the most important contributors to mortality in cancer, and its arrest is likely to lead to meaningful increases in survival time.
